# Small molecule inhibitors of Late SV40 Factor (LSF) abrogate hepatocellular carcinoma (HCC): Evaluation using an endogenous HCC model

**DOI:** 10.18632/oncotarget.4656

**Published:** 2015-07-17

**Authors:** Devaraja Rajasekaran, Ayesha Siddiq, Jennifer L.S. Willoughby, Jessica M. Biagi, Lisa M. Christadore, Sarah A. Yunes, Rachel Gredler, Nidhi Jariwala, Chadia L. Robertson, Maaged A. Akiel, Xue-Ning Shen, Mark A. Subler, Jolene J. Windle, Scott E. Schaus, Paul B. Fisher, Ulla Hansen, Devanand Sarkar

**Affiliations:** ^1^ Department of Human and Molecular Genetics, Virginia Commonwealth University, Richmond, VA 23298, USA; ^2^ Department of Biology, Center for Chemical Methodology and Library Development at Boston University, Boston MA 02215; ^3^ Department of Chemistry, Center for Chemical Methodology and Library Development at Boston University, Boston MA 02215; ^4^ Program in Molecular Biology, Cell Biology, and Biochemistry, Boston University, Boston MA 02215; ^5^ Alnylam Pharmaceuticals, Inc., Cambridge MA 02142; ^6^ Massey Cancer Center, Virginia Commonwealth University, Richmond, VA 23298, USA; ^7^ VCU Institute of Molecular Medicine (VIMM), Virginia Commonwealth University, Richmond, VA 23298, USA

**Keywords:** LSF, HCC, FQI, mitotic arrest, apoptosis

## Abstract

Hepatocellular carcinoma (HCC) is a lethal malignancy with high mortality and poor prognosis. Oncogenic transcription factor Late SV40 Factor (LSF) plays an important role in promoting HCC. A small molecule inhibitor of LSF, Factor Quinolinone Inhibitor 1 (FQI1), significantly inhibited human HCC xenografts in nude mice without harming normal cells. Here we evaluated the efficacy of FQI1 and another inhibitor, FQI2, in inhibiting endogenous hepatocarcinogenesis. HCC was induced in a transgenic mouse with hepatocyte-specific overexpression of c-*myc* (Alb/c-*myc*) by injecting N-nitrosodiethylamine (DEN) followed by FQI1 or FQI2 treatment after tumor development. LSF inhibitors markedly decreased tumor burden in Alb/c-*myc* mice with a corresponding decrease in proliferation and angiogenesis. Interestingly, *in vitro* treatment of human HCC cells with LSF inhibitors resulted in mitotic arrest with an accompanying increase in CyclinB1. Inhibition of CyclinB1 induction by Cycloheximide or CDK1 activity by Roscovitine significantly prevented FQI-induced mitotic arrest. A significant induction of apoptosis was also observed upon treatment with FQI. These effects of LSF inhibition, mitotic arrest and induction of apoptosis by FQI1s provide multiple avenues by which these inhibitors eliminate HCC cells. LSF inhibitors might be highly potent and effective therapeutics for HCC either alone or in combination with currently existing therapies.

## INTRODUCTION

Hepatocellular carcinoma (HCC) is one of the five most common malignancies and the third leading cause of cancer related deaths worldwide [1]. Despite a decline in overall cancer incidence, there is a steady increase in the incidence of HCC, especially in the US [2]. This problem is compounded by the fact that HCC is a disease with very poor prognosis with only 10% of patients having 5 year mean survival rate using currently available treatments [3]. The only FDA-approved drug, sorafenib, for non-resectable HCC provides a survival benefit of only 2.8 months [4]. As such there is an urgent need for developing new molecular targeted therapy for HCC.

We previously identified overexpression of the transcription factor Late SV40 factor (LSF) in ~90% of human HCC patients which could be significantly correlated with the stages of the disease and ‘gain-of-function’ and ‘loss-of-function’ studies established the significance of LSF in promoting hepatocarcinogenesis [5–9]. Furthermore, LSF positively regulates all the major hallmarks of cancer in HCC. First, LSF transcriptionally regulates osteoponin (OPN) in HCC, which activates c-Met signaling, thereby mediating the oncogenic function of LSF and promoting metastasis [5, 6]. Second, we identified matrix metalloproteinase-9 (MMP-9) as a target gene of LSF, important for mediating LSF-induced angiogenesis [8]. Third, LSF overexpression can contribute to chemoresistance [7], since LSF transcriptionally regulates expression of thymidylate synthase [10], a target of the chemotherapeutic agent 5-fluorouracil [7]. Finally, nuclear translocation of LSF upon overexpression of Snail can lead to transcriptional upregulation of fibronectin expression, contributing to induction of the epithelial-mesenchymal transition (EMT) [11].

We previously identified Factor Quinolinone Inhibitors (FQIs) as specific inhibitors of LSF DNA-binding and of LSF transcriptional activation [12]. The prototype, FQI1, markedly inhibited proliferation and induced apoptosis of human HCC cells at low micromolar doses, without causing toxicity to normal immortal human hepatocytes or primary mouse hepatocytes [12]. In a subcutaneous xenograft model of human HCC in athymic nude mice, FQI1 profoundly inhibited tumor growth without causing toxicity to any other organs [12]. These intriguing results suggested that FQIs might be effective therapeutics for HCC, although requiring further stringent evaluation.

We now demonstrate therapeutic efficacy of FQI1 and a related compound FQI2 in an endogenous mouse model of HCC. We also document that FQIs cause mitotic arrest and subsequent apoptosis. The lack of toxicity combined with the targeting of this well-documented vulnerability in cancer cells strongly support further evaluation of FQIs in a Phase I/II clinical trial.

## RESULTS

### Therapeutic efficacy of LSF inhibitors on endogenous HCC

**Figure 1:**
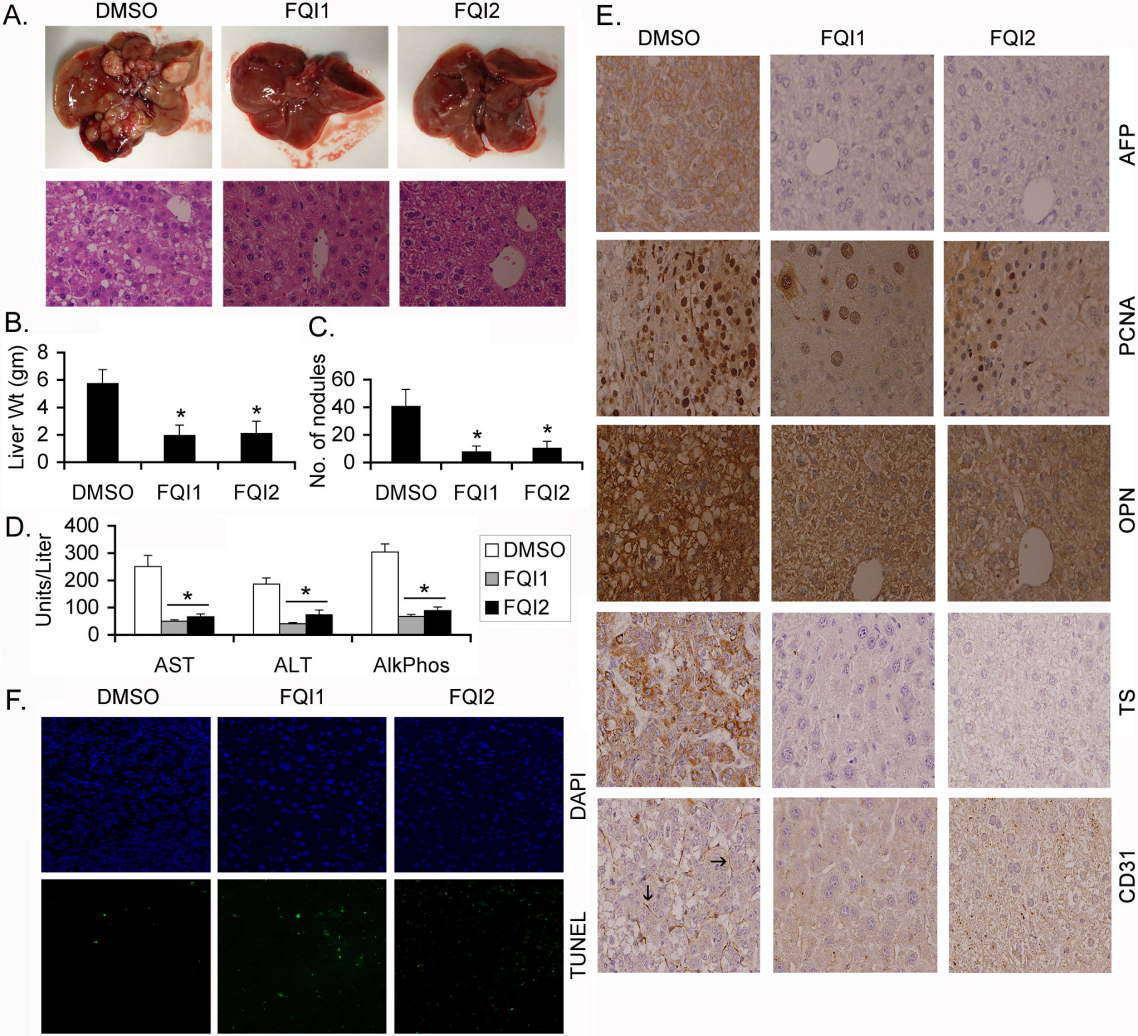
LSF inhibitors abrogate endogenous HCC in Alb/c-myc mice Protocols for induction of HCC and treatment of animals are described in Materials and Methods. **A.** Upper panel, representative photographs of livers of DMSO-, FQI1- and FQI2-treated mice at the end of the experiment. Lower panel, representative H & E stained liver sections of the indicated group at the end of the experiment. Magnification: 400X. **B.** Liver weight of the mice in the indicated treatment groups. **C.** Number of liver nodules in the indicated treatment groups. **D.** Serum levels of aspartate aminotransferase (AST), alanine aminotransferase (ALT) and alkaline phosphatase (Alk Phos) in the indicated treatment groups. For B-D, *n* = 10 in each group. The data represent mean ± SEM. *:*p* < 0.01. **E.** Immunohistochemical analysis of the indicated proteins in the liver sections of the indicated groups. Arrows indicate microvessels. Magnification: 400X. **F.** TUNEL staining in the liver sections of the indicated groups.

Alb/c-*myc* mice spontaneously develop HCC and the kinetics of the hepatocarcinogenic process is significantly accelerated upon treatment with DEN [13]. The chemotherapeutic efficacy of LSF inhibitors was evaluated in Alb/c-*myc* mice harboring DEN-induced liver tumors. The animals, treated with FQI1 and FQI2, demonstrated marked decrease in tumor nodules (2 mm or less in size) when compared to control (vehicle treated) animals (Figure 1A upper panel). Histological examination of the liver showed features of HCC in control animals while FQI1- and FQI2-treated animals maintained normal hepatic architecture (Figure 1A, lower panel). The liver weight (Figure 1B) and number of nodules (Figure 1C) in control mice were significantly higher than that in treated mice suggestive of decrease in tumor burden upon FQI treatment. Biochemically, the level of enzymes indicating liver damage, such as Aspartate Aminotransferase (AST), Alanine Aminotransferase (ALT) and Alkaline Phosphatase, showed significant decreases upon FQI treatment when compared to control (Figure 1D). Immunohistochemical analysis of tumors revealed significant increases in the HCC marker α-fetoprotein (AFP), proliferation marker proliferating cell nuclear antigen (PCNA), LSF target gene osteopontin (OPN) and thymidylate synthase (TS) and angiogenesis marker CD31 only in control animals but not in FQI1- or FQI2-treated animals (Figure 1E). Increased TUNNEL positive cells (apoptotic cells) were observed in FQI1- or FQI2-treated groups when compared to control animals (Figure 1F). No obvious signs of toxicity, such as weight loss or changes in behavior, feeding or grooming, were observed upon FQI1 or FQI2 treatment suggesting that these agents might be potent and non-toxic HCC therapeutics.

### LSF inhibitors decrease proliferation of human HCC cells and induce G_2_/M cell cycle arrest

**Figure 2:**
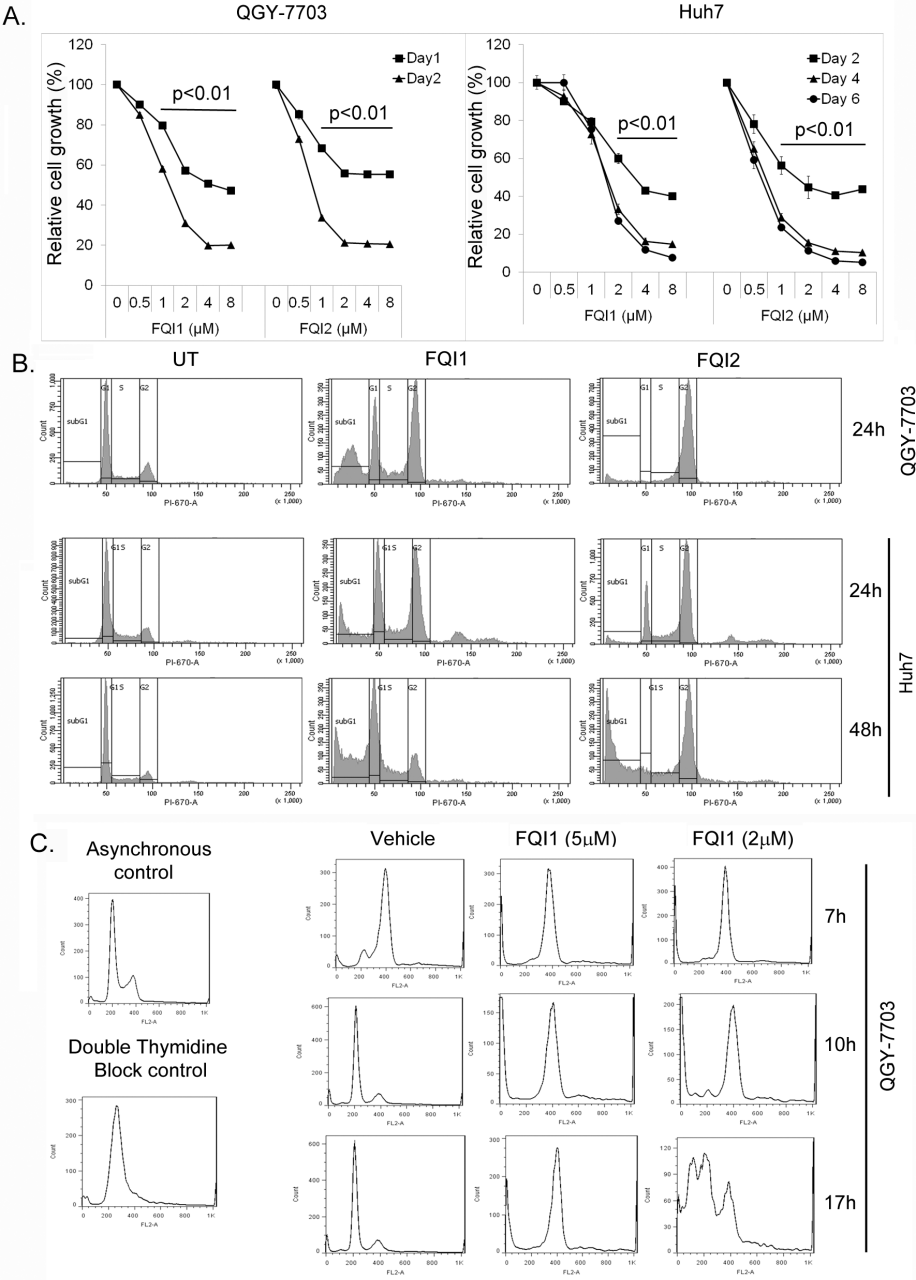
LSF inhibitors cause G2/M arrest **A.** QGY-7703 and Huh7 cells were treated with the indicated concentrations of FQI1 or FQI2 and cell proliferation was determined by standard MTT assay at the indicated time points. The data represent mean ± SEM. *:*p* < 0.01. **B.** Representative cellular DNA content histograms of the indicated cells treated with 2 μM FQI1 or FQI2. UT indicates untreated or vehicle-treated cells. **C.** Representative cellular DNA content histograms of QGY-7703 cells synchronized by double thymidine block and then treated with FQI1 (2 or 5 μM) at the time of release.

To obtain better insights into the mechanism of action of FQI1 and FQI2, we performed a comparative analysis of the effects of these two agents on human HCC cells, QGY-7703 and Huh7. Cell proliferation analysis by standard MTT assay showed that both FQI1 and FQI2 markedly decreased cell growth in a dose- and time-dependent manner (Figure 2A). QGY-7703 cells showed ~90% reduction in cell growth by 48 hours while the kinetics of killing in Huh7 cells was relatively slower. As such for most of the studies we used 24 h treatment for QGY-7703 cells and 48 h treatment for Huh7 cells.

LSF transcriptionally regulates thymidylate synthase and we previously demonstrated that inhibition of LSF in multiple cell types by expression of a dominant negative LSF mutant induces a G1/S block or apoptosis in S phase [10, 14], and in QGY-7703 cells induces cell cycle delay in S phase [15]. To our surprise, treatment of serum-starved and released QGY-7703 and Huh7 cells with 2 μM FQI1 or FQI2 resulted in potent cell cycle arrest in G_2_/M phase along with an increase in sub-G_1_ peak suggestive of apoptosis (Figure 2B). Quantification of distribution of cells in each phase of the cell cycle is provided in Supplementary Figure S1. FQI1 treatment showed an increased sub-G1 peak, compared to FQI2 treatment, in QGY-7703 cells, prompting us to probe into this phenomenon in detail. Since FQI2 is more potent than FQI1 in inhibiting LSF activity and in inhibiting cell proliferation (10), we synchronized QGY-7703 cells at the G_1_/S boundary by double thymidine block and released the cells at 0 h in the presence of FQI1 at 2 μM or 5 μM concentration. Upon analysis of cellular DNA content, vehicle-treated cells re-entered cell cycle in G_1_ phase by 10 h after release, while FQI1-treated cells, with both 2 and 5 μM concentrations, were arrested at G_2_/M phase (Figure 2C). Interestingly, at 17 h post-treatment, cells treated with 5 μM FQI1 maintained G_2_/M arrest while cells treated with 2 μM FQI1 showed an increase in sub-G_1_ peak. These findings suggest that dosage of the drug determines cell fate and that FQI2 exerts a more potent cell cycle inhibitory effect than FQI1, similar to its more potent LSF inhibitory effect.

### Induction of CyclinB1 upon FQI1 and FQI2 treatment

**Figure 3:**
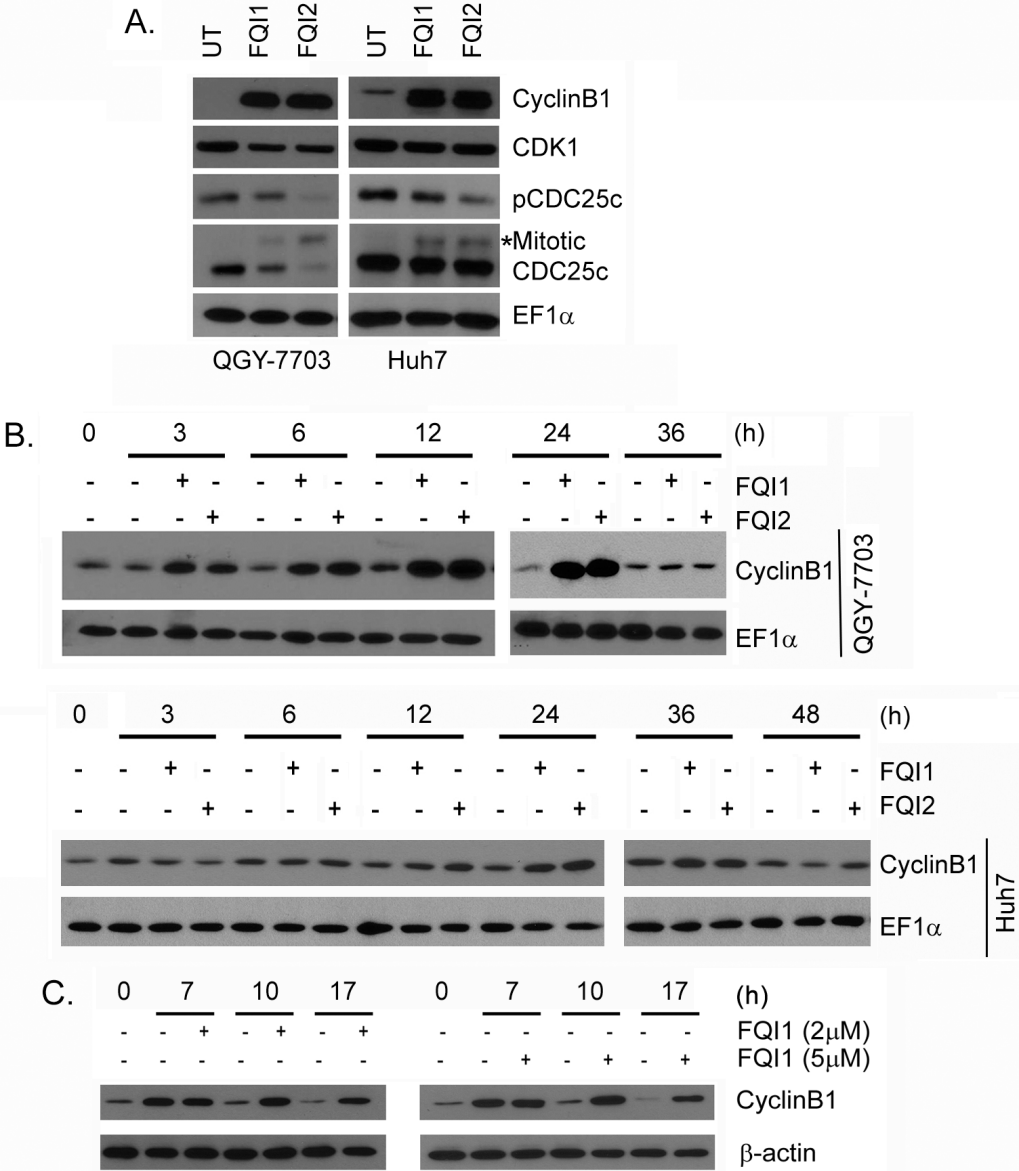
LSF inhibitors augment CyclinB1 levels **A.** Western blot analysis of the indicated proteins in the indicated cells upon treatment with 2 μM FQI1 or FQI2 for 24 h. **B.** Time course analysis of CyclinB1 expression level in the indicated cells upon treatment with FQI1 or FQI2. EF1α expression was analyzed as loading control. **C.** Time course analysis of CyclinB1 expression level in QGY-7703 cells synchronized by double thymidine block and then treated with FQI1 (2 or 5 μM) at the time of release.

We checked the expression pattern of CyclinB1, CDK1 and CDC25c, which function at the G_2_/M transition, upon FQI1 and FQI2 treatment. CyclinB1 level was significantly induced upon FQI1 and FQI2 treatment in HCC cell lines (Figure 3A). While the level of CDK1 remained unchanged, an increase in the mitotic form of CDC25c with corresponding decrease in phospho-Ser216-CDC25c was observed upon FQI1 and FQI2 treatment (Figure 3A).

The increase in CyclinB1 upon FQI treatment was analyzed in more detail using a time-course analysis. Upon release of serum-starved cells, CyclinB1 induction was evident as early as 3 h after FQI1 treatment in QGY-7703 cells and at 12 h post-treatment in Huh7 cells with peak induction observed at 24 h for QGY-7703 cells and 36 h for Huh7 cells (Figure 3B). Immunofluorescence analysis confirmed increased expression of CyclinB1 in FQI-treated QGY-7703 cells compared to control cells (Supplementary Figure S2). Nuclear translocation of CyclinB1 was observed at 12 h of FQI treatment especially with FQI2 (Supplementary Figure S2). To determine whether the kinetics of increase in Cyclin B1 levels corresponded to the normal kinetics at the G_2_/M transition, CyclinB1 expression levels were determined in QGY-7703 cells synchronization by a double thymidine block and addition of 2 μM or 5 μM FQI1 at the time of release. At 7 h post-release, both vehicle-treated and FQI-treated cells showed an increase in Cyclin B1 levels. At 10 h post-treatment, when vehicle-treated cells returned to G_1_ (Figure 2C), CyclinB1 level returned to base-line level, as expected. In contrast, the increased level of cyclin B1 was sustained in FQI1-treated cells (Figure 3C).

### Inhibition of CyclinB1 or CDK1 protects from G_2_/M arrest upon FQI treatment

**Figure 4:**
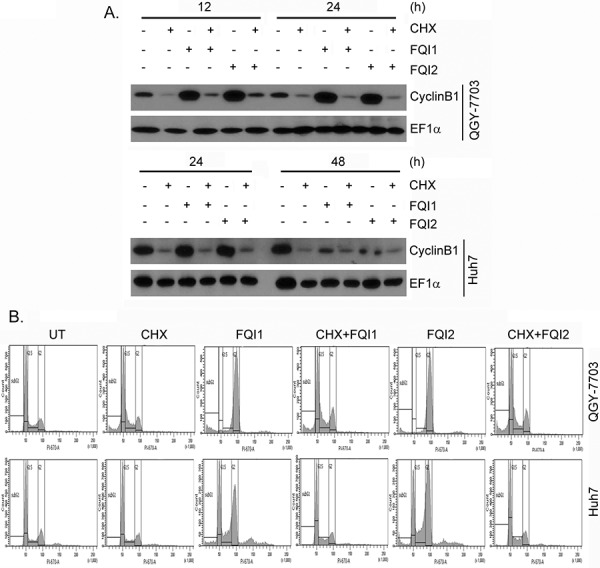
Cycloheximide (CHX) treatment protects HCC cells from FQIs-induced G2/M arrest **A.** Western blot analysis of CyclinB1 and EF1α in the indicated cells upon 2 h pre-treatment with CHX followed by treatment with FQI1 or FQI2. **B.** Representative cell cycle histograms of the indicated cells upon 2 h pre-treatment with CHX followed by treatment with FQI1 or FQI2.

**Figure 5:**
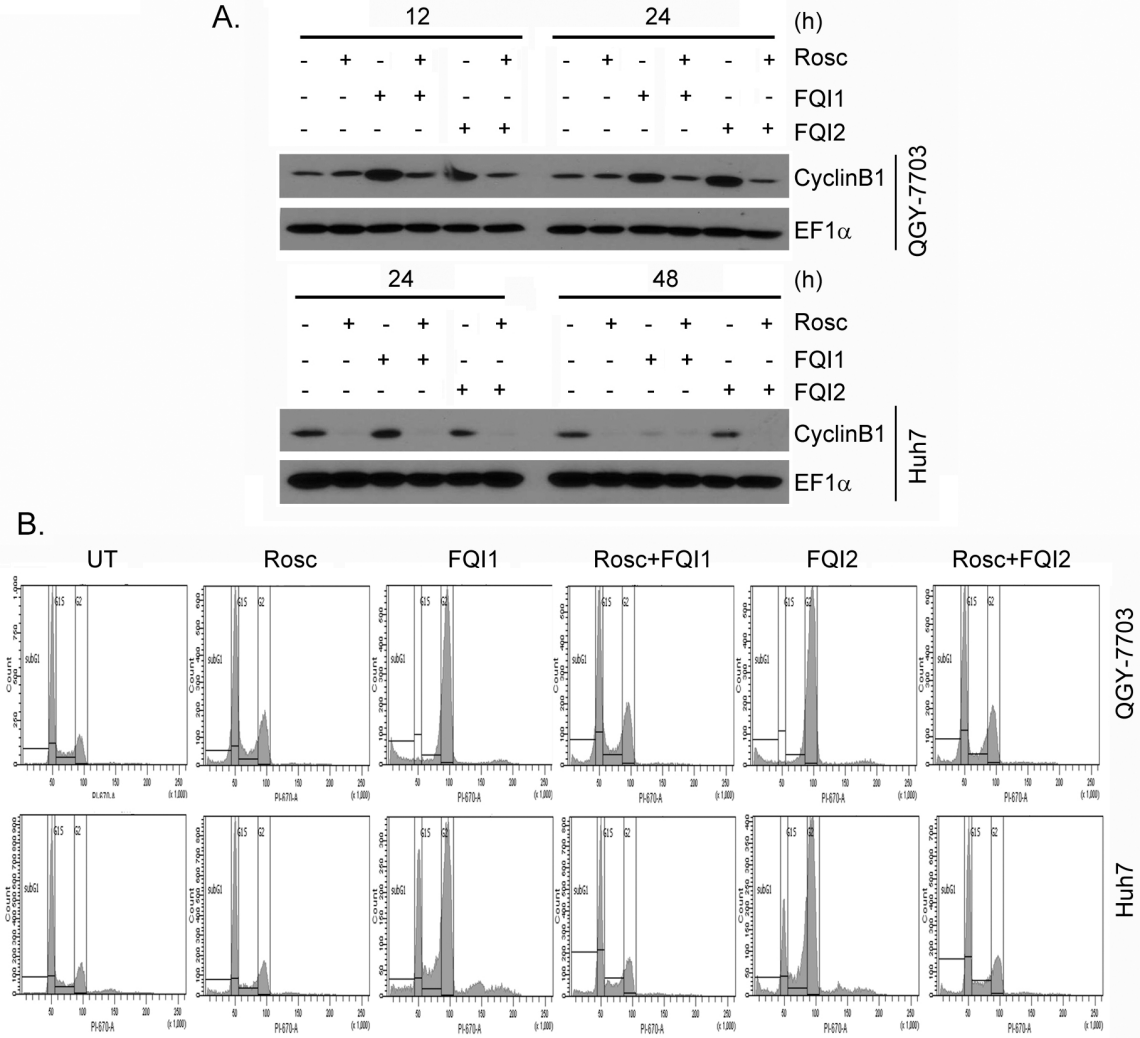
Inhibition of CDK1 protects HCC cells from FQIs-induced G2/M arrest **A.** Western blot analysis of CyclinB1 and EF1α in the indicated cells upon 2 h pre-treatment with Roscovitine (Rosc) followed by treatment with FQI1 or FQI2. **B.** Representative cell cycle histograms of the indicated cells upon 2 h pre-treatment with Roscovitine (Rosc) followed by treatment with FQI1 or FQI2.

Since *de novo* protein synthesis is required for CyclinB1 induction at G_2_/M checkpoint we treated the cells with protein synthesis inhibitor cycloheximide (CHX; 10 μg/ml) and performed cell cycle analysis. CHX treatment markedly inhibited CyclinB1 induction upon FQI treatment, as expected (Figure 4A). Correspondingly a profound protection from FQI-induced G_2_/M arrest was observed upon CHX treatment (Figure 4B and Supplementary Figure S3). Inhibition of CDK activity by treatment with a cell-permeable inhibitor Roscovitine (Rosc; 30 μM) also abrogated CyclinB1 induction upon FQI treatment (Figure 5A) and provided marked protection from G_2_/M arrest (Figure 5B and Supplementary Figure S4). These findings suggest a key role of CyclinB1/CDK1 in the maintenance of G_2_/M arrest upon FQI treatment. The role of CyclinB1 was checked by transfecting CyclinB1 siRNA and analyzing cell cycle following FQI treatment. CyclinB1 siRNA provided significant protection from FQI-induced cell cycle arrest (Supplementary Figure S5). However, the magnitude of the protection was not as robust as that provided by CHX or Roscovitine which might be due to long kinetics of the assay using the siRNA.

Persistent CyclinB1 overexpression has been identified as a marker for recurrent HCC [16]. We, therefore analyzed DMSO and FQI-treated tumor samples for CyclinB1 expression. While low level CyclinB1 expression was detected in vehicle-treated tumors, it was barely detectable in FQI-treated normal liver tissue (Supplementary Figure S6). These findings suggest that proliferating tumor cells express low level of CyclinB1. In FQI-treated cells, transient high induction of CyclinB1 in tumor cells leads to cell cycle arrest and eventual death. As such at the end of the study CyclinB1 expression is not detected in FQI-treated normal liver tissue.

### LSF inhibitors induce mitotic arrest

**Figure 6:**
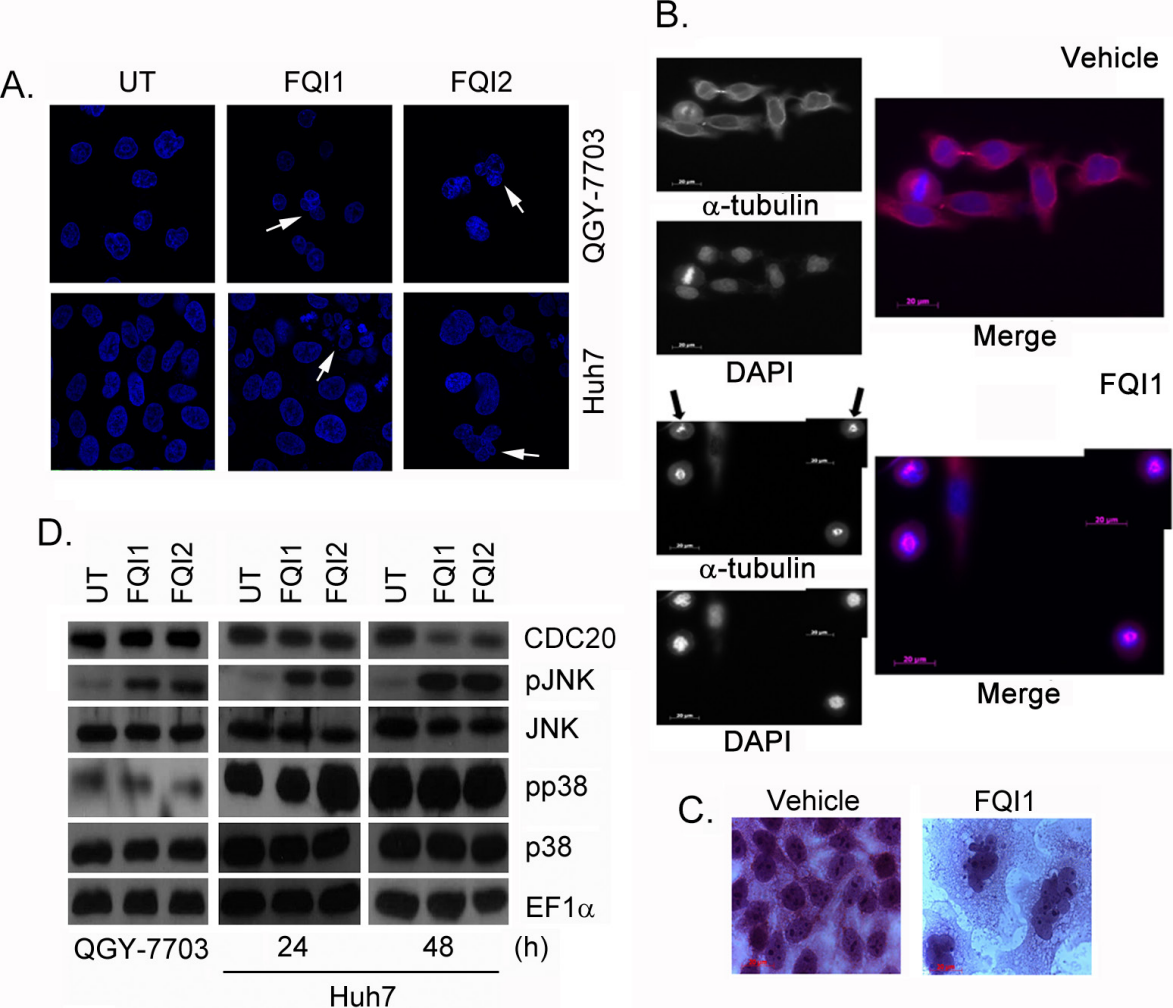
FQIs induce mitotic arrest. QGY-7703 cells were treated for 12 h while Huh7 cells were treated for 24 h with FQIs **A.** Representative photomicrographs of DAPI-stained cells. Arrows indicate multinucleated cells. **B.** QGY-7703 cells were synchronized by double thymidine block and then released in the presence of 2 μM FQI1. Cells harvested at 11 h post-release were stained for α-tubulin and DNA (using DAPI). Representative images taken at 63x magnification are shown. **C.** QGY-7703 cells were treated as in B except with 5 μM FQI1. Cells were stained with Hematoxylin and Eosin 48 h post-release. **D.** Western blot analysis of the indicated proteins in the indicated cells upon treatment with FQI1 or FQI2. QGY-7703 cells were treated for 24 h.

The induction of mitotic CDC25c upon FQI treatment (Figure 3A) suggests potential mitotic arrest of the cells. Staining of DNA in QGY-7703 and Huh7 cells with DAPI clearly showed multinucleated cells upon FQI treatment, which result from slippage after mitotic arrest (Figure 6A, arrows). To capture the bulk of the cells shortly after FQI-mediated cell cycle blockage, QGY-7703 cells, synchronized by double thymidine block and released in the presence of 2 μM FQI1, were harvested at 11 h post-release. Cells were co-stained for α-tubulin and DNA (using DAPI). Most of the cells in the vehicle group have already undergone mitosis, although a few were imaged still at metaphase or undergoing cytokinesis (Figure 6B). In contrast, the FQI-treated cells remained in prometaphase with condensed, but not congressed, DNA and spindle that were either incomplete or disrupted (the latter indicated with arrows) (Figure 6B). At later time-points (48 h), multinucleation was also observed in H&E-stained QGY-7703 cells (Figure 6C). A sustained activation of stress-activated kinase JNK, but not p38 MAPK, was observed when the cells were treated with FQIs (Figure 6D).

### LSF inhibitors induce apoptosis

**Figure 7:**
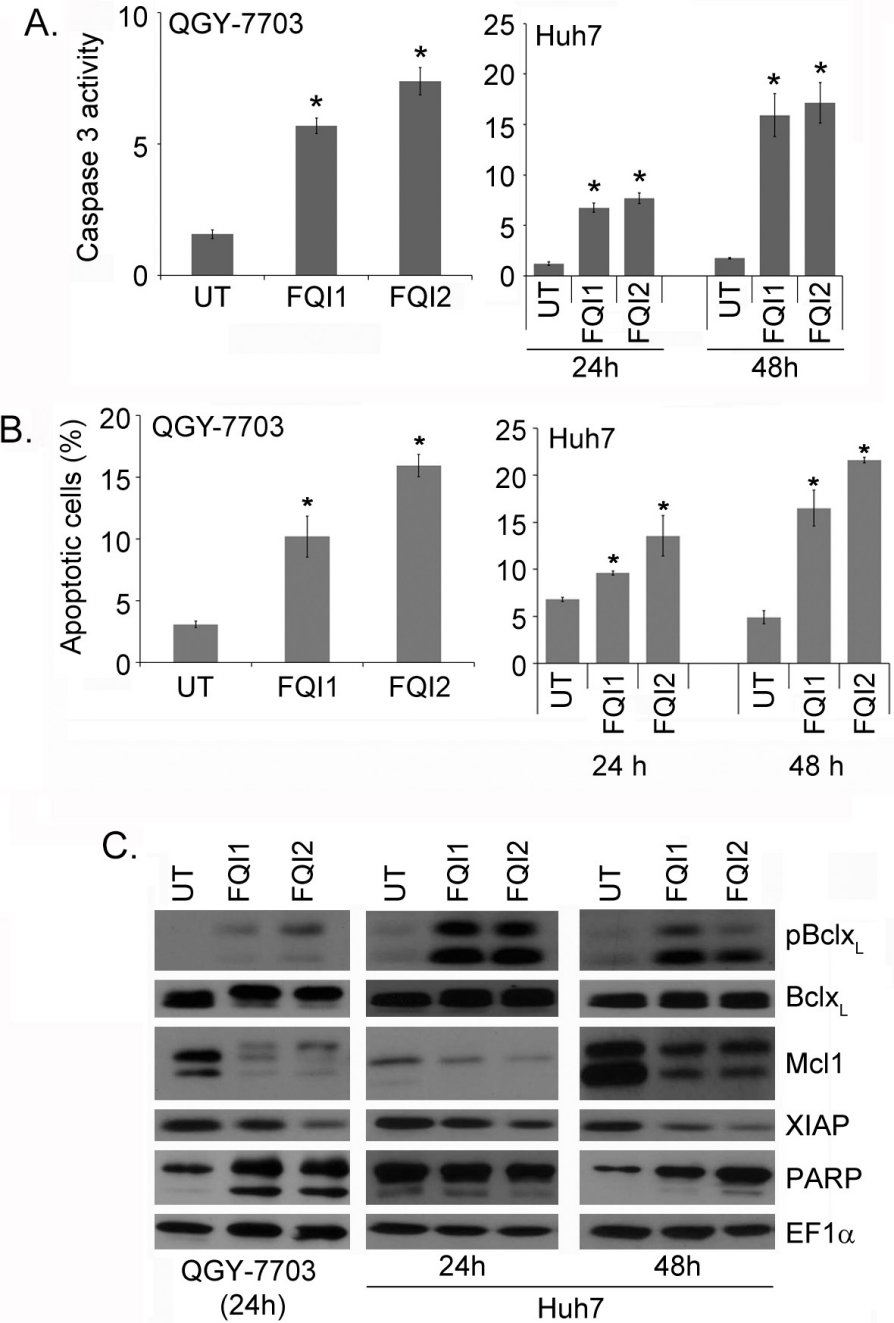
LSF inhibitors induce apoptosis **A.** Caspase 3 activity (mM of AMC released/mg protein/30 min) was measured in the indicated cells upon treatment with 2 μM FQI1 or FQI2. QGY-7703 cells were treated for 24 h. The data represent mean ± SEM. *:*p* < 0.01. **B.** Apoptosis was measured by Annexin V staining followed by flow cytometry in the indicated cells upon treatment with 2 μM FQI1 or FQI2. QGY-7703 cells were treated for 24 h. The data represent mean ± SEM. *:*p* < 0.01. **C.** Western blot analysis of the indicated proteins in the indicated cells upon treatment with FQI1 or FQI2 for the indicated periods of time.

We previously showed that FQI1 treatment of QGY-7703 leads to apoptosis, as measured by TUNEL staining [12]. To further these findings in the context of the mitotic phenotypes, we characterized this apoptotic cell death upon FQI treatment using a number of other molecular markers. In QGY-7703 and Huh7 cells a significant increase in Caspase 3 activity was observed upon treatment with FQI1 or FQI2 (Figure 7A). Correspondingly, a significant increase in apoptotic cells was observed upon Annexin V staining followed by flow cytometry (Figure 7B). Cleavage of PARP, another marker of apoptosis, was observed in these cells when treated with FQI (Figure 7C). Although the total level of the anti-apoptotic protein Bcl-x_L_ did not change, an increase in phospho-Bcl-x_L_ and a decrease in the anti-apoptotic proteins Mcl1 and XIAP were observed in the HCC cells upon treatment with LSF inhibitors (Figure 7C).

## DISCUSSION

In the present study we report the chemotherapeutic efficacy of LSF inhibitors on DEN-induced HCC in Alb/c-*myc* transgenic mice. We previously documented that the FQI1 inhibits human HCC xenografts in a nude mouse model [12]. Here we document that treatment with LSF inhibitors significantly reduced tumor burden in Alb/c-*myc* mice accompanied by a significant decrease in serum levels of liver enzymes that are markers of liver damage [17]. In addition both FQI1 and FQI2 significantly inhibited proliferation and angiogenesis and caused a significant decrease in the expression of the LSF-target gene OPN [5]. These results confirm that FQIs maintain their functional attributes, which were initially gleaned from *in vitro* and nude mice studies [12], in an immunocompetent endogenous HCC model. It should be noted that the treatment with FQIs was initiated at 22 weeks when tumors have already developed suggesting that FQIs caused tumor regression rather than just inhibition of growth. Further *in vitro* assays demonstrate that FQIs exert a broad spectrum of anti-cancer effects involving mitotic arrest, which significantly bolsters their clinical relevance as HCC therapeutics.

The transition of cells from G_2_ to M phase of cell cycle is controlled by members of the Cyclin/CDK family [18]. The activation of CyclinB1/CDK1 is required for progression of cells from G_2_ to M Phase in normal cells [19]. The progression of cells to M phase requires stepwise activation of CDK1 [20]. CDK1 can be deactivated by phosphorylation at Thr14 and Tyr15 by Wee1 and Myt1 [21, 22]. Cdc25C is a phosphatase that dephosphorylates Thr14 and Tyr15 of CDK1. Cdc25C can be inactivated by phosphorylation at Ser216 by Chk1 and subsequent sequestration of Cdc25C in the cytoplasm by binding of Chk2 with members of the 14-3-3 protein family prevents premature mitosis [23]. Many cytotoxic compounds and DNA damaging agents are known to arrest cells in different stages of the cell cycle and prevent proliferation of cancer cells. These compounds often either deregulate or inhibit the activation of Cyclin/CDKs resulting in cell cycle arrest. We demonstrate that treatment with LSF inhibitors induced a pronounced G_2_/M phase arrest with a significant increase in CyclinB1. We also observed an increase in the activity of Cdc25c phosphatase and decreased levels of p-Ser216-Cdc25C which is an inactive form of Cdc25c. This will in turn activate CDK1 leading to activation of CyclinB1/CDK1 complex. Our results therefore suggest that LSF inhibitors induce G_2_/M phase cell cycle arrest, consistent with sustained activity of the CyclinB1/CDK1 complex.

To check whether upregulation of CyclinB1/CDK1 complex is symptomatic of FQI-mediated mitotic blockage and apoptosis, we inhibited cell cycle progression with either roscovitine or cycloheximide. Treatment with Roscovitine, a synthetic CDK inhibitor, has been shown to induce a partial G_2_ cell cycle arrest in MCF7 cells [24, 25]. We document that treatment with Roscovitine along with LSF inhibitors greatly inhibited FQI-induced upregulation of CyclinB1 expression. Flow cytometric analysis showed that there was slight accumulation of cells in the G_2_/M phase in Roscovitine-treated cells when compared to control cells. However, Roscovitine plus FQI1 treatment resulted in the same amount of G2/M phase cells, therefore indicating that Roscovitine inhibited the mitotic defect caused by FQI. Treatment with CHX prevented Cyclin B1 induction, as expected, and also prevented the FQI-mediated G_2_/M phase defects. These results indicate that induction of G_2_/M phase arrest by LSF inhibitor, which is marked by upregulation of CyclinB1/CDK1 activity, requires cell cycle progression into mitosis.

Prolonged activation of CyclinB1/CDK1 during mitotic arrest activity leads to phosphorylation of numerous apoptotic regulatory proteins such as Mcl1, Bcl-2, Bcl-x_L_, Caspase-9, Caspase-8, and stabilization of survivin and/or maintenance of XIAP expression [26, 27]. Our results show that mitotic arrest upon treatment with LSF inhibitors leads to sustained elevation of CyclinB1/CDK1, with increased phosphorylation and degradation of Mcl1 and Bcl-x_L_. The activity of JNK was also increased which might mediate hyperphosphorylation and degradation of Mcl1. Furthermore, XIAP expression was decreased upon LSF inhibitor treatment. Proapoptotic indicators, such as increased Caspase-3 activity and PARP cleavage, were also observed. These findings provide a mechanistic basis for why cells undergo apoptosis after prolonged mitotic arrest induced by LSF inhibitors.

In summary, we document that the FQI family of LSF inhibitors have multiple consequences. On one hand, as previously shown, inhibition of LSF transcriptional activity blocks induction of LSF target genes that mediate downstream oncogenic signaling. Additionally, these inhibitors cause mitotic arrest culminating into apoptosis. These multiple targets exert profound anti-cancer activity which is confirmed by marked reduction in tumor burden in DEN-treated Alb/c-*myc* mice. The mechanism by which LSF inhibitors induce mitotic arrest remains to be fully elucidated, and is a focus of current efforts. Lack of toxicity combined with multimodal anti-cancer mechanisms support the hypothesis that LSF inhibitors may prove to be clinically relevant and potentially effective therapeutics for HCC. Since LSF promotes both primary tumors and metastasis FQIs might be useful for treating advanced metastatic HCC either alone or in combination with receptor tyrosine kinase inhibitors, such as sorafenib, thereby providing dual pronged attack for successful elimination of the disease.

## MATERIALS AND METHODS

### Chemicals

Small molecule inhibitors of LSF, FQI1 and its achiral analogue FQI2, have been described previously [12]. After initial titration, *in vitro* assays were performed using either 2 or 5 μM FQI1 or FQI2. As indicated, cells were pre-treated for 2 h with Cycloheximide (10 μg/ml; Sigma; C7698) or Roscovitine (30 μM; Cell Signaling; #9885) before treatment with LSF inhibitors. Control and Cyclin B1 siRNAs were obtained from Santa Cruz Biotech (sc-37007 and sc-29284, respectively).

### Animal studies

The generation and characterization of Alb/c-*myc* mouse have been described previously [13]. These mice were kindly provided by Dr. Snorri S. Thorgeirsson (NIH/NCI). Wild-type mice were bred with heterozygous Alb/c-*myc* mice to obtain the experimental cohort of animals. For induction of chemical carcinogenesis, a single intraperitoneal (i.p.) injection of 10 μg/gm body weight of N-nitrosodiethylamine (DEN) was given at 14 days of age to male Alb/c-*myc* mice [13]. The animals were divided randomly into 3 groups (10 mice per group) and treated with i.p. injections of FQI1 or FQI2 (4 mg/kg). The treatment started at 22 weeks of age with 3 cycles of treatment (5 injections per week per cycle) over 6 weeks and the animals were sacrificed 2 weeks after the last injection. Blood samples were collected for liver enzyme analysis at the Molecular Diagnostic Laboratory, Department of Pathology, Virginia Commonwealth University. The liver tissue was fixed in 10% formalin and used for histopathology and immunohistochemistry studies.

### Cells, culture condition, cell cycle and apoptosis assays

Human HCC cells, QGY-7703 and Huh7, were cultured as previously described [12, 28]. Cell proliferation was determined by standard MTT assays as described [12, 28]. In initial experiments, HCC cells were synchronized by culturing in growth medium containing 0.5% FBS for 48 h, followed by FQI1 or FQI2 treatment in complete growth medium containing 10% FBS. For Cycloheximide and/or Roscovitine experiments, serum-starved synchronized cells were pretreated with respective agents for 2 h in complete growth medium prior to FQI1 or FQI2 treatment. Additionally, cell cycle synchronization of QGY-7703 cells was achieved by double thymidine block essentially as described previously [29], followed by release at 0 h in the presence of FQI1. At the end of the experiment cells were harvested, fixed in 70% ethanol and stained with propidium iodide followed by flow cytometry for cell cycle analysis [30]. For siRNA experiments, QGY-7703 cells were synchronized by serum starvation for 24 h following which siRNAs were transfected. After 24 h, the cells were again synchronized by overnight serum starvation, treated with FQI1 or FQI2 for 24 h and cell cycle was analyzed. Apoptosis was determined by annexin V-binding assay followed by flow cytometry as described [31]. The activity of Caspase-3 was assessed by the EnzChek caspase-3 assay kit from Molecular Probes using the manufacturer's protocol.

### Preparation of whole cell lysates and western blot analyses

Preparation of whole-cell lysates and Western blot analyses were performed as described [28]. The primary antibodies used were anti-CyclinB1 (1:1,000, rabbit polyclonal; Cell Signaling), anti-Cyclin B1 (1:1000, mouse monoclonal [V152]; Abcam), anti-CDK1 (1:1, 000, rabbit polyclonal; Cell Signaling), anti-pSer216-CDC25c (1:1,000, rabbit monoclonal; Cell Signaling), anti-CDC25c (1:1,000, rabbit monoclonal; Cell Signaling), anti-p-JNK (1:1,000, rabbit monoclonal; Cell Signaling), anti-JNK (1:1,000, rabbit polyclonal; Cell Signaling), anti-p-p38 MAPK (1:1,000, rabbit monoclonal; Cell Signaling), anti-p38 MAPK (1:1,000, rabbit monoclonal; Cell Signaling), anti-p-Bcl-x_L_ (1:1,000, rabbit polyclonal; Thermo Scientific), anti-Bcl-x_L_ (1:1,000, rabbit monoclonal; Cell Signaling), anti-Mcl1 (1:1,000, rabbit monoclonal; Cell Signaling), anti-XIAP (1:1,000, rabbit polyclonal; Cell Signaling) and anti-PARP (1:1,000, rabbit polyclonal; Cell Signaling). Blots were stripped and normalized by reprobing with anti-EF1α (1:2,000, mouse monoclonal; Millipore) and β-actin (1:20,000, mouse monoclonal; Sigma).

### Immunohistochemistry

Immunohistochemistry was carried out using formalin-fixed paraffin-embedded (FFPE) sections as described [28]. The sections were blocked in phosphate buffered saline-tween 20 (PBST) using 10% normal goat serum. The primary antibodies were diluted in PBST containing 5% goat serum. The primary antibodies used were: anti-AFP (Santa Cruz; rabbit polyclonal; 1:50); anti-CD31 (Dako; mouse monoclonal; 1:50); anti-PCNA (Cell Signaling; mouse monoclonal; 1:100); anti-OPN (Millipore; rabbit polyclonal; 1:500); anti-CyclinB1 (1:100, rabbit polyclonal; Cell Signaling). Secondary antibodies were diluted in PBST containing corresponding 2.5% blocking serum. The signals were developed by avidin-biotin-peroxidase complexes with a DAB substrate solution (Vector laboratories). Images were analyzed using an Olympus microscope.

### Immunofluorescence

Immunofluorescence was performed as described [28]. The primary antibody was anti-α-Tubulin (Cell Signaling; mouse monoclonal; 1:2000) and the secondary antibody was Alexa488-conjugated anti-mouse IgG (Molecular Probes; 1:500). The slides were mounted in VectaShield fluorescence mounting medium containing 4, 6-diamidino-2-phenylindole (Vector Laboratories). Images were analyzed using a Zeiss confocal laser scanning microscope. Alternatively, cells were stained with α-tubulin antibody (1:50, mouse monoclonal; Abcam) followed by a CY5-conjugated secondary anti-mouse antibody (1:200; Abcam). The cover slips were mounted with Invitrogen Anti Fade Mounting Medium containing DAPI and imaged using a Zeiss AxioImager.Z1 microscope at 63x magnification.

### Immunocytochemistry

QGY-7703 cells synchronized with a double thymidine block were released in the presence of 5 μM FQI1. Forty-eight hours after release, cells were harvested, fixed, and stained using Hematoxylin and Eosin. Images were captured using a Zeiss AxioImager. Z1 microscope at 100x magnification.

### Statistical analysis

Data were represented as the mean ± Standard Error of Mean (S.E.M) and analyzed for statistical significance using one-way analysis of variance (ANOVA) followed by Newman-Keuls test as a post hoc test. A *P* value of < 0.05 was considered as significant.

## SUPPLEMENTARY FIGURES



## Abbreviations

LSF: Late SV40 Factor
FQI: Factor Quinolinone Inhibitor
MMP-9: Matrix Metalloproteinase 9
OPN: Osteopontin
DEN: N-nitrosodiethylamine
AFP: α-fetoprotein
PCNA: proliferating cell nuclear antigen
AST: Aspartate Aminotransferase
ALT: Alanine Aminotransferase
