# Supplementary material for: Small molecule inhibitors of Late SV40 Factor (LSF) abrogate hepatocellular carcinoma (HCC): Evaluation using an endogenous HCC model
Source: Oncotarget. 2015 Jul 17;6(28):26266–77. doi: 10.18632/oncotarget.4656 (PMC4694900; doi:10.18632/oncotarget.4656)
Supplement: Supplementary file 1 [file oncotarget-06-26266-s001.pdf]

SUPPLEMENTARY FIGURES

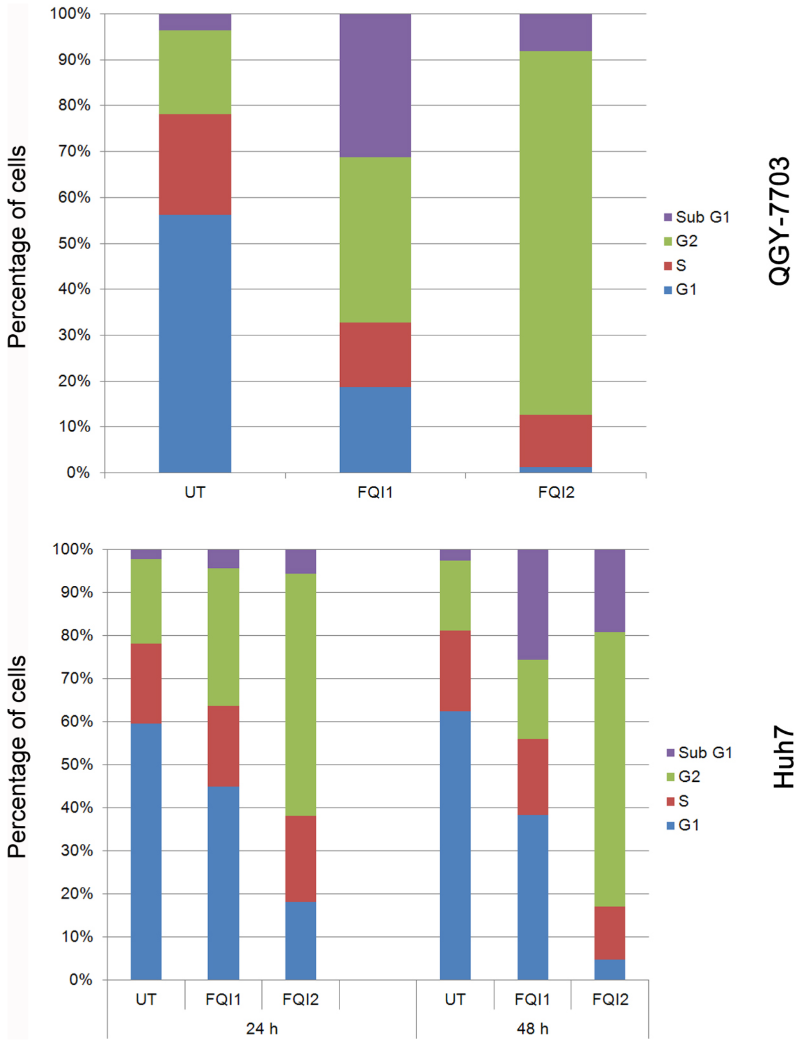

Supplementary Figure S1: LSF inhibitors cause G2/M arrest. Graphical representation of percentage of cells in different phases of cell cycle upon treatment with FQI1 or FQI2.

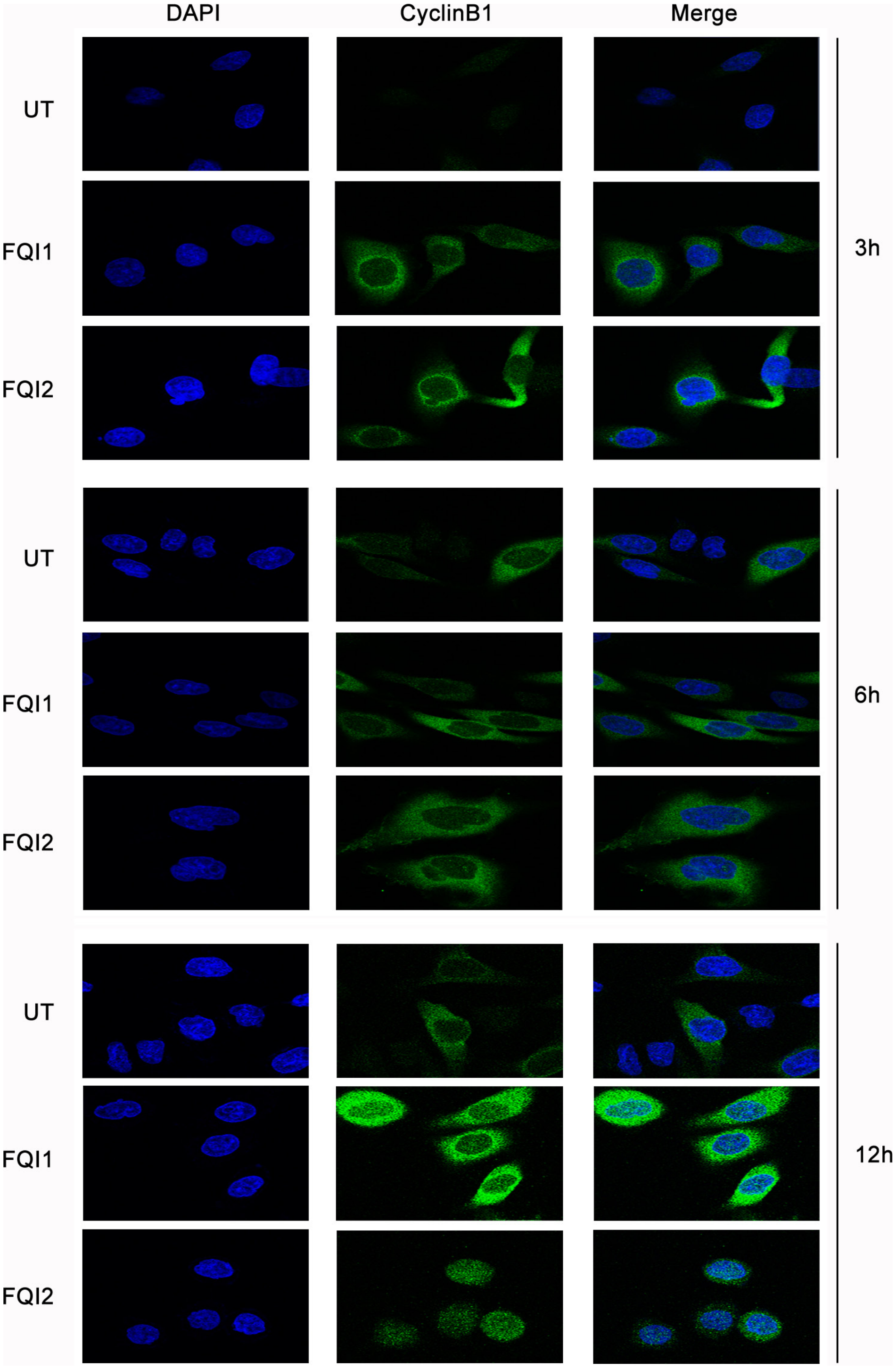

Supplementary Figure S2: LSF inhibitors induce CyclinB1 leading to its nuclear accumulation. Representative photomicrograph of QGY-7703 cells either untreated (UT) or treated with FQI1 or FQI2 and stained for CyclinB1. The images were taken by a Zeiss confocal laser scanning microscope.

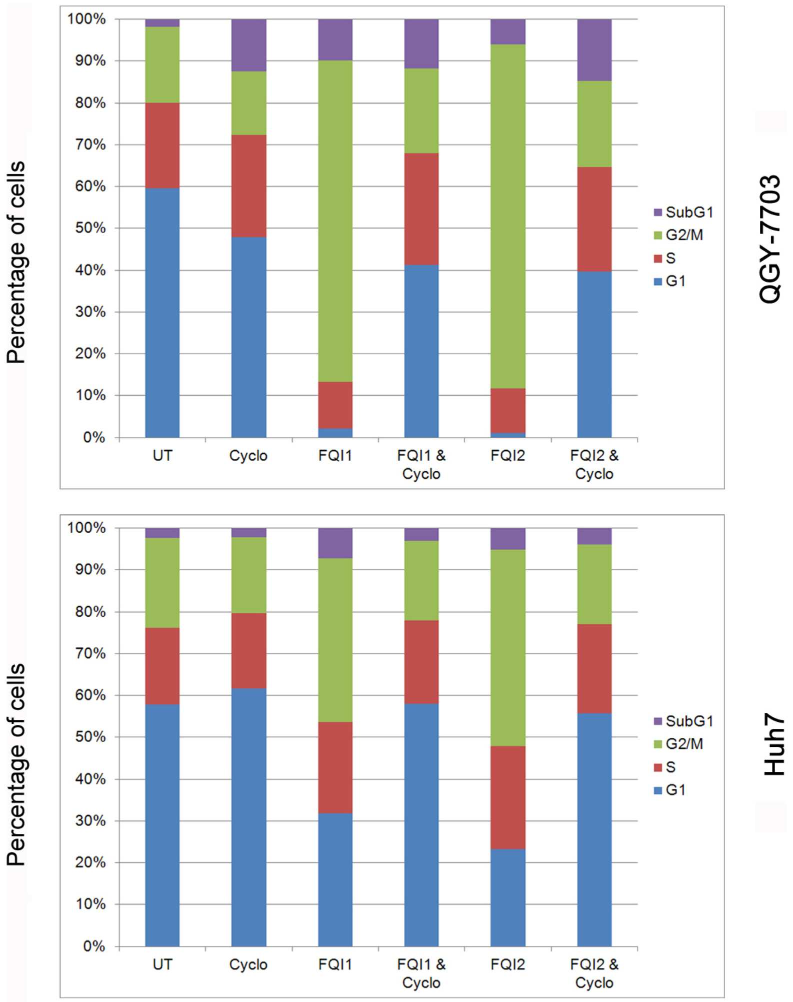

Supplementary Figure S3: Cycloheximide (CHX) treatment protects from FQIs-induced G2/M arrest. Graphical representation of percentage of cells in different phases of cell cycle upon pre-treatment with CHX followed by treatment with FQI1 or FQI2.

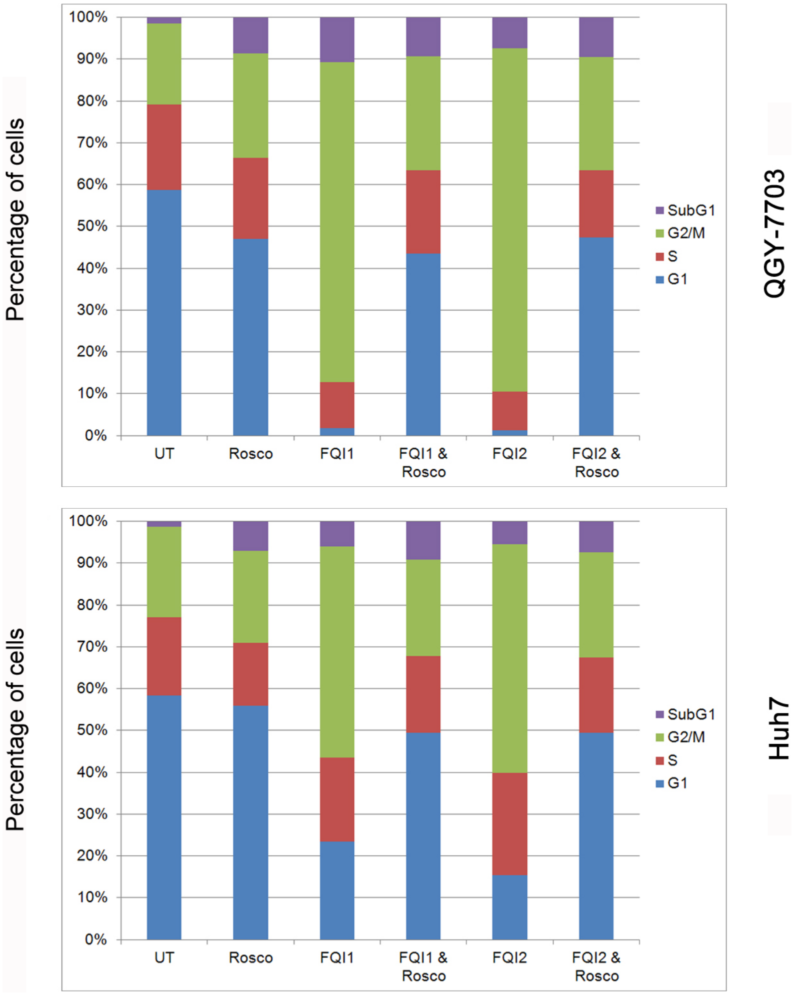

Supplementary Figure S4: Inhibition of CDK1 protects from FQIs-induced G2/M arrest. Graphical representation of percentage of cells in different phases of cell cycle upon pre-treatment with Roscovitine followed by treatment with FQI1 or FQI2.

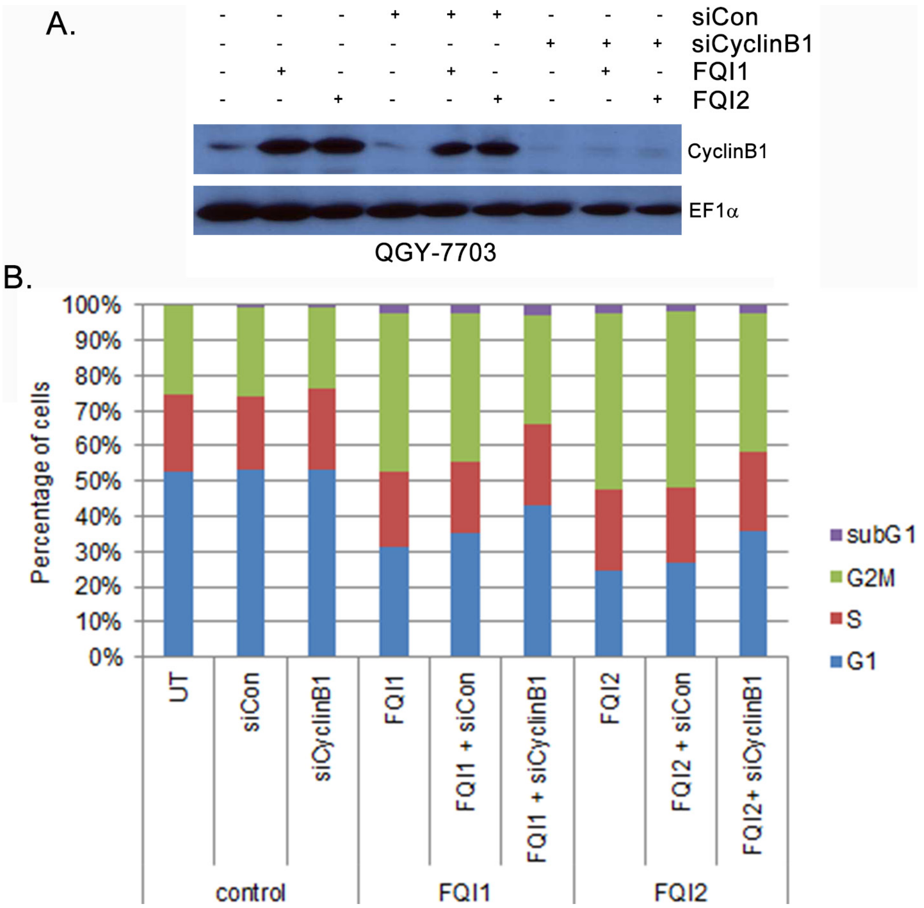

Supplementary Figure S5: Downregulation of CyclinB1 by siRNA partially protects from FQIs-induced G2/M arrest in QGY-7703 cells. QGY-7703 cells were synchronized with 0.5% FBS for 24 h. Control scrambled siRNA (siCon) and CyclinB1 siRNA (siCyclinB1) were transfected and 24 h following transfection cells were further synchronized with 0.5% FBS overnight and then treated with DMSO, FQI1 or FQI2 for 24 h. **A.** Western blot analysis to detect CyclinB1 levels. FQI treatment increases the levels of CyclinB1 which was blocked with siCyclinB1. **B.** Graphical representation of percentage of cells in different phases of cell cycle.

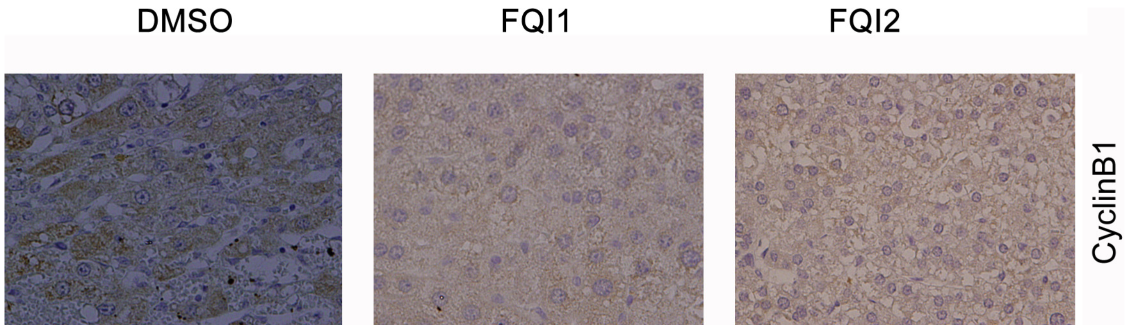

Supplementary Figure S6: CyclinB1 expression is not detected in FQI-treated normal liver. Immunohistochemical analysis of CyclinB1 in the liver sections of the indicated groups. Magnification: 400X.
